# Iron-Doped Hydroxyapatite Nanoparticles for Magnetic Guided siRNA Delivery

**DOI:** 10.3390/ijms26167712

**Published:** 2025-08-09

**Authors:** Hina Inam, Lorenzo Degli Esposti, Federico Pupilli, Marta Tavoni, Francesca Casoli, Simone Sprio, Anna Tampieri

**Affiliations:** 1 Institute of Science, Technology and Sustainability for Ceramics (ISSMC), National Research Council of Italy (CNR), 48018 Faenza, Italy; hina.inam@issmc.cnr.it (H.I.); federico.pupilli@issmc.cnr.it (F.P.); marta.tavoni@issmc.cnr.it (M.T.); 2 Department of Material Science and Technology, University of Parma, 43121 Parma, Italy; 3 Dipartimento di Chimica e Chimica Industriale, Università Degli Studi di Genova, 16146 Genova, Italy; lorenzo.degliesposti@unige.it; 4 Institute of Materials for Electronics and Magnetism (IMEM), National Research Council of Italy (CNR), 43124 Parma, Italy; francesca.casoli@imem.cnr.it

**Keywords:** magnetic hydroxyapatite, siRNA delivery, nanomedicine

## Abstract

Small interfering RNAs (siRNAs) are particularly attractive among the frontier drugs due to their high specificity of action, activity on disease-inducing genes, and small molecular weight, thus being one of the most studied agents for gene therapy. However, siRNAs are prone to fast enzymatic degradation in the bloodstream, as well as other limitations that challenge their clinical translation. Nanoparticle (NP) delivery of siRNA has been proposed as a potential solution, overcoming their intrinsic limitations. In this regard, the siRNA delivery by magnetic nanoparticles is of particular interest because, being susceptible to external magnetic fields, it may be guided remotely, maximizing transfection efficiency and minimizing side effects. In addition, magnetic NPs would also allow a theranostic combination of drug delivery, magnetic resonance imaging, and hyperthermia. In this work we have studied the uptake of a model therapeutic siRNA by iron-doped hydroxyapatite nanoparticles (FeHA NPs), which are known to have excellent biocompatibility and magnetic susceptibility. We discovered that FeHA NPs stabilized by citrate (Cit-FeHA NPs) uptake siRNA by adsorption quickly and with high efficiency (ca. 90%) without altering nanoparticles physicochemical properties or colloidal stability. SiRNA-loaded Cit-FeHA NPs are able to slowly release their payload, with a sustained release of 45 days without siRNA degradation. Our work is therefore the preliminary validation of the suitability of FeHA NPs for magnetically guided delivery of therapeutic siRNAs.

## 1. Introduction

The use of small interfering RNAs (siRNAs) as therapeutic agents is becoming more and more relevant for the treatment of a wide range of diseases, including cancer, viral infections, and genetic disorders [1,2]. SiRNAs induce sequence-specific gene silencing through RNA interference, downregulating the expression of disease-related genes [2]. SiRNAs are a promising therapeutic approach, able to treat congenital disorders as well as specific target sites not achievable by conventional molecular drugs or antibodies. Furthermore, their synthesis is relatively non-complicated in comparison to antibodies or protein-based drugs [3]. In recent decades, significant progress has been made in siRNA-based therapeutics, leading to the approval of siRNA-based therapeutic agents [4].

Therapeutic methods involving monoclonal antibodies and small-molecule inhibitors have challenges due to their multi-target range and difficulties in finding specific agents, while siRNAs have better specificity in a sequence-dependent way. Among the various interference RNAs (RNAi), regulatory non-coding RNAs (ncRNAs) can either stimulate or inhibit the expression of target genes, their aberrant expression being an essential component in the pathogenesis of many human disorders. In several pathological situations, the expression or action of specific genes is compromised, and miRNAs become aberrant in several diseases, such as hepatitis, cardiac diseases, and cancer. Hence, RNAi can be used therapeutically to provide transcription-suppressive variables, kinases, and other signaling molecules to recover the expression of these dysfunctional genes. Several siRNA copies could regulate target gene expression in a single target cell, via complementary base pairing, providing benefits over conventional therapeutics. The RNAi-based approach also benefits from advancements in whole-genome sequencing, establishing a robust basis for medication development and personalized therapies. RNAi treatments have shown effectiveness also in cancer therapy by targeting oncogenes, tumor suppressor genes, and other regulatory genes, with the potential to inhibit cancer angiogenesis and address the problem of drug resistance induced by inhibitors [5].

However, the translation and clinical application of siRNAs is hindered by several delivery-related challenges. These include short lifetime in the bloodstream due to degradation by nucleases, limited cellular uptake by passive diffusion, rapid renal clearance, and potential stimulation of the immune system [6]. To overcome these obstacles, research efforts have been focused on siRNA delivery systems for protecting them and facilitating their delivery to target tissues and cells [6,7].

The successful clinical translation of RNAi therapies relies on delivery systems that efficiently carry ncRNAs to their site of action with very low side effects, enhance cellular uptake, and facilitate targeted delivery. Different kinds of nanomaterial-based delivery systems have been designed to improve the therapeutic effectiveness of RNA interference. Nanoparticles (NPs) have been used as siRNA delivery systems. NPs can be designed to withhold and protect siRNAs from degradation and can also be functionalized to enhance cellular uptake, provide targeted delivery, and have a controlled release [7].

Lipid-based nanoparticle (LNP) systems are the most advanced non-viral carriers for RNA interference delivery, thanks to their biocompatibility, minimal immunogenicity, and inherent capacity for effective interaction with cell membranes facilitating cellular uptake. In addition to lipid nanoparticles (LNPs), which are prevalent in clinical applications, other noteworthy platforms are: (i) cyclodextrin-containing polymers to produce stable complexes with siRNA and enhance endosomal escape; (ii) polymeric nanoparticles like polyethyleneimine (PEI) with high siRNA loading efficiency and potential for surface functionalization; and (iii) dendrimers, as a class of highly structured three-dimensional macromolecule that allow for precise size regulation and multivalent binding, thereby enhancing cellular uptake and reducing off-target effects [8].

An interesting implementation of NP delivery is the use of magnetic NPs for magnetic guidance. These NPs, whose most common representative are superparamagnetic iron oxide NPs, are susceptible to external magnetic fields and can be directed to specific tissues non-invasively by using external magnetic signals (magnetic guidance) [9,10]. This external control allows for site-specific accumulation, further enhancing therapeutic efficacy. Moreover, magnetic NPs can be tracked in vivo using magnetic resonance imaging, enabling theranostic applications [11]. Recently it has been demonstrated that magnetic guidance of siRNA-loaded magnetic NPs significantly improves transfection efficiency in vitro and in vivo [12,13].

In vivo research indicates that a magnetic core can assess the target site and size of cancer in vivo by magnetic resonance imaging. Magnetic particles have shown efficacy in monitoring treatment outcomes, particularly the inhibition of tumor growth [14,15].

RNAi imaging may also monitor target cells, so indirectly confirming the efficiency of gene silencing. A study introduced a nano-sized theranostic agent with magnetic nanoparticles, an MRI reporter labeled with Cy5.5 dye for near-infrared fluorescence (NIRF) imaging, and linked to siRNA targeting specific genes for islet transplantation in the treatment of type 1 diabetes [16].

However, iron oxide-based magnetic NPs need to be functionalized extensively to become biocompatible and to avoid aggregation and immune clearance. Most importantly, these NPs have raised toxicity concerns both for acute toxicity as well as for accumulation in organs such as liver and kidneys [17,18]. To have a biocompatible and magnetically responsive NP, we have developed the iron-doped hydroxyapatite nanoparticles (FeHA NPs) [19]. FeHA NPs possess the excellent biocompatibility of hydroxyapatite NPs, well known for their excellent biocompatibility, bioactivity, and osteoconductivity, as well as superparamagnetic behavior conferred by simultaneous Fe^3+^/Fe^2+^ co-doping in Ca(I) and Ca(II) calcium sites [20]. FeHA is highly biocompatible and has been used for drug delivery enhanced by magnetic guidance, magnetic resonance imaging, and hyperthermia [19,21,22].

The aim of the present work is to assess preliminarily the suitability of FeHA NPs as nano-vehicles for magnetic-guided delivery of therapeutic siRNAs. We have enhanced FeHA NPs colloidal stability by a surface functionalization with citrate ions (referred to as Cit-FeHA NPs) and studied their capability to absorb and release a model positive control siRNA.

## 2. Results

### 2.1. Characterization of Pure Hydroxyapatite, FeHA, and Citrate-FeHA Nanoparticles

The synthesis of FeHA nanoparticles has been conducted by modulating relevant process parameters such as reaction temperature, pH, and dropping speed. The purpose of this modulation was to obtain an Fe-doped apatitic phase with maximized magnetization extent but preserve bioactivity by limiting the formation of magnetite as a secondary phase causing cytotoxicity. Reaction temperature was fixed at 45 °C as a compromise between the formation and crystallization of Fe-doped apatite and to limit magnetite formation, which is detected at T > 40 °C [19,20]. Reaction pH was kept at ~9 during the whole synthesis to prevent the formation of undesired acidic CaP phases such as CaHPO_4_·2H_2_O (brushite). In addition, we limited reagent dropping speed to 3 mL/min parallel to vigorous stirring to maintain the forming suspension in a well-homogenized medium, preventing the formation of local acidic regions where pH can drop below ~6 and induce the nucleation of brushite. Afterwards, FeHA was surface-functionalized with citrate (Cit-FeHA) to enhance its colloidal stability and to impart a strongly negative surface charge.

**Figure 1:**
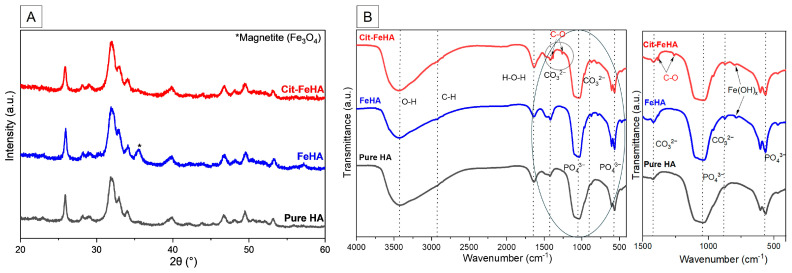
(**A**) PXRD patterns and (**B**) FTIR spectra of FeHA and Cit-FeHA in comparison to HA. In right panel there is a magnification of the 1500–400 cm^−1^ spectral region (circled section of panel (**B**)) where most of the IR absorption bands are present.

The PXRD analysis (Figure 1A) of FeHA and Cit-FeHA in comparison to the control HA confirms that apatite is the main crystalline phase in both samples, as all diffraction peaks correspond to tabulated values (JCPDS reference pattern 00-009-0432). Magnetite phase in the form of nanoparticles was detected as a secondary phase in FeHA in an amount estimated as ~9 wt.%. HA, FeHA, and Cit-FeHA show broad diffraction peaks indicating low crystallinity extent, ascribed to reduced crystallite size related to the low synthesis temperature [23]. The functionalization of FeHA with citrate did not alter the material’s crystallinity (Table S1); however, it affected the phase composition. Indeed, in Cit-FeHA the magnetite phase is absent, as shown by the PXRD diffraction pattern. A purification effect of citrate can be hypothesized: citrate acts as a surface functionalization agent, linking FeHA nanoparticles as well as magnetite nanoparticles. As citrate-stabilized magnetite nanoparticles are smaller than citrate-stabilized FeHA nanoparticles, they have a stronger resultant buoyancy force, and the two can be separated by centrifugation.

Structural characterization was also performed by FTIR spectroscopy (Figure 1B). All samples exhibited the characteristic vibrational band of apatite, i.e., the main mode ν_3_PO_4_ as a broad band centered at ca. 1047 cm^−1^, as well as the double-split band at 595 and 567 cm^−1^ of ν_4_PO_4_ mode [24]. The presence of carbonate ions in the samples was confirmed by the observation of characteristic vibrational bands in the FTIR spectra. In particular, a weak band at approximately 874 cm^−1^ corresponds to the ν_2_ bending mode of the CO_3_^2−^ ion, while two weak bands at around 1415 and 1455 cm^−1^ are attributed to the asymmetric stretching modes (ν_3_) of carbonate. These features are consistent with the B-type carbonate substitution, where carbonate ions replace phosphate groups in the apatite lattice. According to the literature, B-type substitution is typically identified by the appearance of a ν_2_ carbonate band near 872–874 cm^−1^ and ν_3_ bands in the range of 1410–1470 cm^−1^, while the presence of A-type can be confirmed by the presence of the ν_3_ band around 1540 cm^−1^ (associated with CO_3_^2−^ replacing OH^−^). In our samples, no significant absorption near 1540 cm^−1^ was observed, further supporting the predominance of B-type substitution [25,26]. Additional band at 1641 cm^−1^ related to adsorbed water (ν_H2O_ mode) [24]. In comparison to HA, FeHA presents additional bands at 802 cm^−1^, due to Fe(OH)_x_ species; in detail, this band is associated with entrapped water hydroxyls and librational modes of nonstoichiometric OH units, respectively [20]. The spectrum of Cit-FeHA confirms the presence of adsorbed citrate, as additional bands are observed at 1260 and 1380 cm^−1^ due to symmetric and antisymmetric stretching of the C-O bond in the carboxylate groups of citrates (ν_COO_ mode) [27].

The chemical composition of the materials is reported in Table 1. ICP-OES confirms the presence of iron in the FeHA sample. The actual doping of iron into the apatitic structure was confirmed by subtracting the Fe amount due to magnetite, thus finding that the total iron content in the FeHA phase is ~10 mol% (~6.9 wt.%), i.e., about half of the nominal iron introduced in the reaction vessel (20 mol %). Another confirmation of Fe doping comes from the Ca/P molar ratio, which is lower than the typical stoichiometric value of HA (1.67), indicating a decrease in calcium content due to Fe substitution in the apatite lattice. However, the (Fe + Ca)/P molar ratio of FeHA, purged from the contribution of magnetite (coded as (Ca+Fe_HA_)/P), is 1.69, very close to the theoretical one, confirming the stoichiometric doping of iron in calcium sites. Considering the evaluation of the Fe^2+^ content by titration and UV-Vis quantification, the Fe(III)/Fe(II) molar ratio is much above the nominal value of 1, which means that Fe^3+^ is the most prominent iron species in FeHA. This predominance may be due to the oxidation of Fe^2+^ to Fe^3+^ during the synthetic process. The total content of iron in Cit-FeHA is much lower than in the non-functionalized FeHA. This is consistent with the removal of magnetite during the functionalization with citrate. Interestingly, the Fe^2+^ content remains comparable for the two materials (~0.7 wt.%), whereas the Fe^3+^/Fe^2+^ ratio slightly decreases in Cit-FeHA, thus suggesting that part of the Fe^3+^ ions have been removed during the FeHA stabilization with citrate.

Table 1 also reports the specific surface area of the materials (SSA_BET_) determined by nitrogen gas adsorption. The SSA_BET_ values of HA and FeHA are comparable (~100 m^2^/g) and relatively high, in agreement with the nanometric nature of the materials. Cit-FeHA exhibited a significantly higher surface area (~200 m^2^/g), more than double that of FeHA. This significant increase is attributed to the surface functionalization by citrate molecules, which decreases particle-particle aggregation and hence increases the specific surface area.

**Figure 2:**
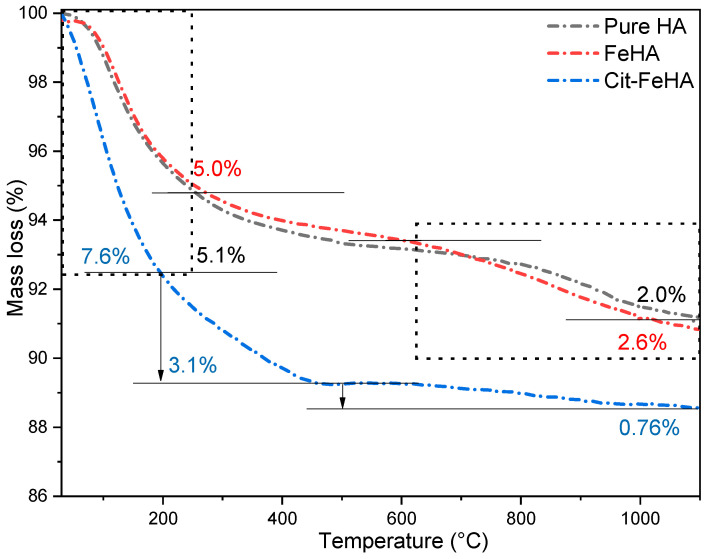
Thermogravimetric curves of Cit-FeHA, FeHA, and HA.

The content of volatile species was assessed by TGA analysis (Figure 2 and Table 2). HA and FeHA show two mass losses, one between room temperature and 250 °C, attributed to removal of adsorbed and structural water, and one between 600 and 1100 °C related to decomposition of structural carbonate ions [20]. Water and carbonate content are comparable for these two materials. On the other hand, Cit-FeHA shows more prominent mass losses at low temperatures as well as an additional weight loss between 250 and 500 °C. This mass loss was attributed to decomposition of adsorbed citrate [28], which was quantified as ca. 3.1 wt.%. On the contrary, carbonate content is lower in comparison to FeHA. These changes indicate that citrate adsorption induced a structural change in the surface layers of FeHA nanocrystals, increasing adsorbed water content while decreasing carbonate content.

**Figure 3:**
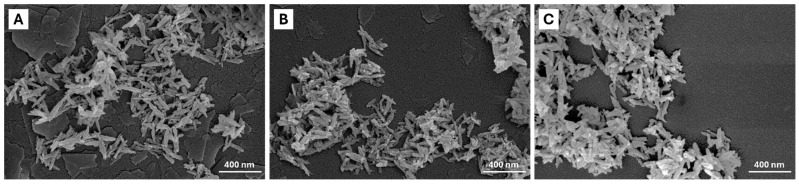
FE-SEM micrographs of (**A**) HA, (**B**) FeHA, and (**C**) Cit-FeHA.

The morphology of the materials was observed by FE-SEM (Figure 3), showing in all materials an elongated needle-like morphology. To further inspect such characteristics, dimensional analysis was carried out (see Supplementary Information, Figure S1). Morphological analysis (reported in Table 3) shows that iron doping does not substantially influence the morphological features of the resulting NPs, as seen by comparing FeHA length, width, and aspect ratio with respect to the HA counterpart. Moreover, citrate functionalization does not lead to an increase in NP size.

To further inspect how iron doping influences the colloidal stability of the samples prepared, DLS and electrophoretic mobility measurements were assessed (Table 3 and Figure S2). All materials possess a narrow, monomodal particle size distribution with an average hydrodynamic diameter ranging from 170 to 220 nm and low PdI. Cit-FeHA has the lowest Z-average and PdI values, confirming that citrate functionalization improves nanoparticle dispersibility and decreases aggregation in aqueous medium. Hydrodynamic diameters of the samples are correlated to their surface charge. Indeed, HA and FeHA present a negative ζ-potential of −18 and −24 mV, respectively, while Cit-FeHA has a strongly negative charge of −34.6 mV. These data prove that, as expected, citrate functionalization enhanced FeHA surface charge due to the presence of exposed negatively charged carboxylate groups on the Cit-FeHA surface, incrementing the electrostatic repulsion between nanoparticles and, thus, their colloidal stability.

**Figure 4:**
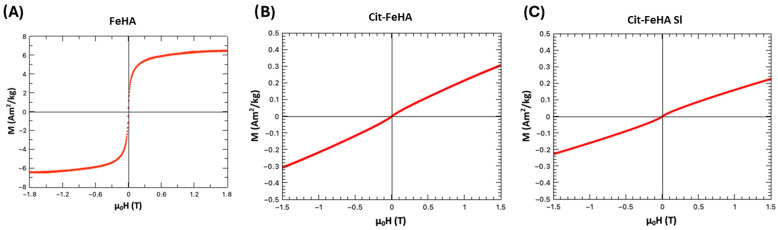
Magnetic hysteresis curves in function of the applied field at T = 300 °K for (**A**) FeHA, (**B**) Cit-FeHA, and (**C**) Cit-FeHA-Sl.

The analysis of magnetic properties by AGFM reports on positive magnetic susceptibility of Fe-HA and anhysteretic magnetization curves with superparamagnetic/paramagnetic behavior and a saturation of magnetization at ~7 Am^2^/kg. (Figure 4). Magnetic measurements carried out on citrate-functionalized FeHA reveal that the magnetization is strongly decreased as the magnetite secondary phase is removed, whereas the material retains a superparamagnetic/paramagnetic behavior with a prominence of the paramagnetic contribution. As previously reported, paramagnetic contribution to FeHA magnetic properties is related to the presence of Fe^3+^ ions in the apatite lattice [19]. Therefore, we can conclude that the functionalization with citrate is appropriate for the removal of iron oxide secondary phases while retaining a mild magnetization extent suitable for magnetic guidance. After loading Cit-FeHA with siRNA (see below) its magnetization is not significantly affected, indicating that its magnetic properties are not hampered by its payload.

### 2.2. Cit-FeHA Nanoparticle In Vitro Cyocompatibility Assay

**Figure 5:**
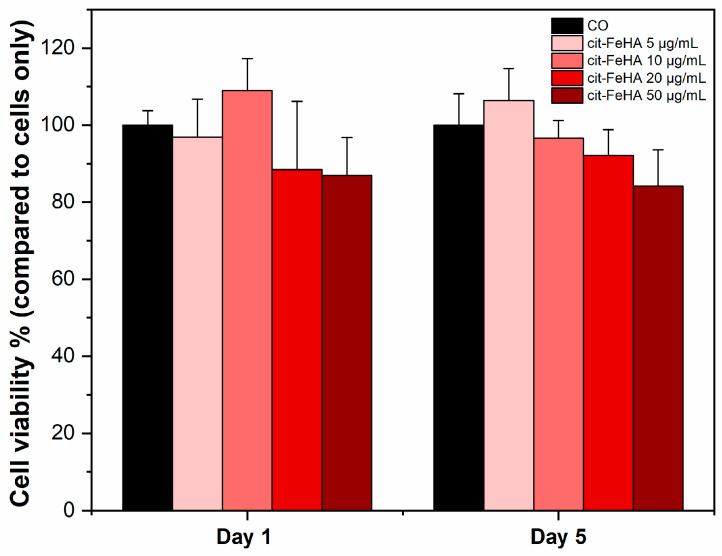
Cell viability analysis. MTT assay was performed after one day and five days of WS1 cells culture with 50 µg/mL, 20 µg/mL, 10 µg/mL, and 5 µg/mL of Cit-FeHA nanoparticles. Cells only (C.O.) were used as control (mean ± standard error).

The MTT assay results (in Figure 5) were carried out to validate the cytocompatibility of Cit-FeHA nanoparticles within a concentration range designed to replicate those commonly employed in in vitro siRNA delivery studies. This range was deliberately selected to represent authentic working conditions in cell culture systems [29]. Cell viability results highlighted minimal cytotoxicity of the nanoparticles. Although minimal dose-dependent cytocompatibility was observed, particularly on day five of NPs incubation, statistical analysis indicated no significant differences in cell viability between treated and control groups (*p* > 0.05), implying that exposure to the FeHA nanoparticles did not negatively impact metabolic activity under the tested conditions. The absence of significance further reinforces the appropriateness of these nanoparticles for siRNA delivery, as sustaining cellular health is essential for maintaining transfection efficiency and subsequent gene regulation results.

### 2.3. Characterization of siRNA-Loaded Cit-FeHA NPs and Release Properties

SiRNA was loaded onto Cit-FeHA NPs by adsorption. Two different NP concentrations were tested, i.e., a higher concentration more suited for administration in small quantities (4 mg/mL, referred as “high” concentration) and a lower one, which may reduce particle–particle interactions and, thus, promote siRNA adsorption (1 mg/mL, referred as “low” concentration). For this preliminary work, we have used a commercial positive control siRNA, which targets GAPDH mRNA, as a model for therapeutic siRNA. At 4 mg/mL NP concentrations siRNA binding efficiency was at 82% with a payload of 0.18 nmol/mg, while in the lower Cit-FeHA concentration of 1 mg/mL both yield (87%) and payload (0.59 nmol/mg) increased (Table 4). This indicates that lowering NP concentration likely decreased surface saturation or aggregation effects, which limits the availability of active sites for siRNA adsorption. Regarding the colloidal stability of siRNA-functionalized NPs, it can be observed that siRNA adsorption led to a small increase in the hydrodynamic diameter and PdI paralleled to a decrease in ζ-potential. These variations are expected, as part of the surface carboxylic moieties are involved in siRNA binding. However, the decrease in colloidal stability is minimal and does not compromise NP properties. For these reasons, it was decided to further study the material adsorbed at 1 mg/mL.

**Figure 6:**
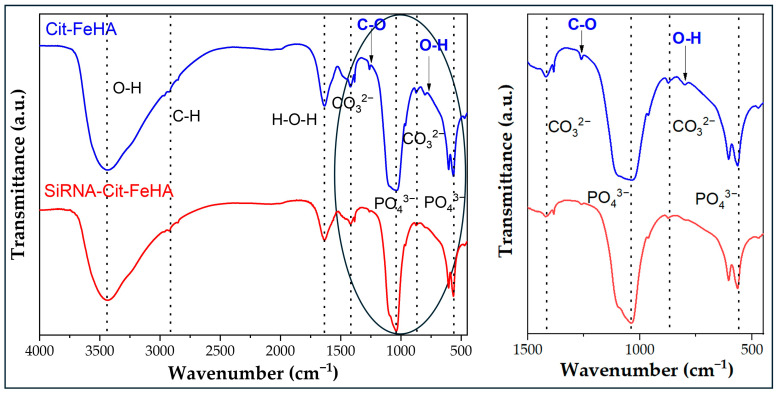
FTIR spectra of Cit-FeHA-Sl in comparison to Cit-FeHA. Cit-FeHA-Sh FTIR spectrum is identical to Cit-FeHA-Sl. In right panel B there is a magnification of the 1500–400 cm^−1^ spectral region (see circled section in Panel A) where most of the IR absorption bands are present.

The FTIR spectrum of both siRNA-Cit-FeHA samples in comparison to Cit-FeHA (Figure 6) confirms that siRNA adsorption did not alter the apatitic structure of the materials nor the citrate content. The adsorbed siRNA cannot be observed through this technique, as its amount is below instrumental sensitivity.

**Figure 7:**
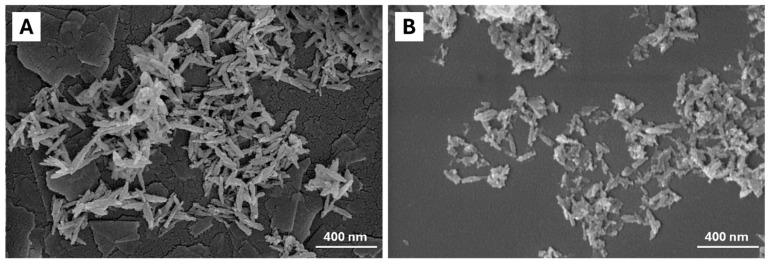
FE-SEM micrographs of (**A**) Cit-FeHA, and (**B**) Cit-FeHA-Sl. Cit-FeHA-Sh FE-SEM micrographs show a particle morphology identical to Cit-FeHA-Sl.

FE-SEM micrographs of siRNA-Cit-FeHA (Figure 7) show a particle size and morphology comparable to Cit-FeHA. However, small variations in surface texture and particle distribution may be observed, probably attributed to surface modification caused by the adsorption of siRNA. In detail, siRNA-FeHA nanoparticles are more porous and aggregated and show surface roughness and irregularities.

**Figure 8:**
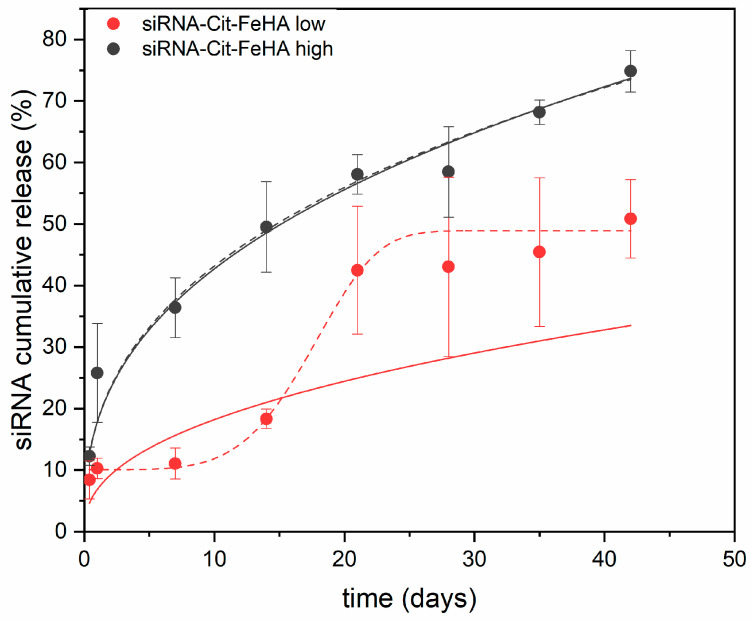
siRNA release curves from siRNA-Cit-FeHA samples, along with the fitted curves using the Korsmeyer–Peppas model (solid line) and the Weibull model (dotted line).

The capability of Cit-FeHA to withhold and release siRNA over time was assessed by studying their release kinetics in RNAse-free water for both high- and low-concentration materials (Figure 8). Release curves collected over 6 weeks show an initial burst release of siRNA over 24 h accounting for ~25% for Cit-FeHA-Sh and 12% for Cit-FeHA-Sl. Such an initial burst release is probably due to the desorption of weakly adsorbed siRNA, thus suggesting that the high concentration of Cit-FeHA NPs not only hindered siRNA adsorption but also led to a weaker interaction. We can hypothesize that in such a condition the particle-particle interaction limited the direct linking of siRNA to the Cit-FeHA NPs, forming instead multiple, less tightly bound siRNA layers.

In the following weeks the two samples showed a similar sustained release over time. At the end of the experiment, after 6 weeks, ~70% and 50% of total siRNA has been released by Cit-FeHA-Sh and Cit-FeHA-Sl, respectively, suggesting that the remaining payload can be released in an even longer timeframe. Overall, both siRNA-functionalized samples show the potential to retain and deliver therapeutic siRNA over a long period of time. Release mechanism kinetics was further explored by fitting the siRNA cumulative release data. Among the models tested, Weibull and Korsmeyer–Peppas (KP) model showed the best fit to the siRNA release data, indicating their suitability for describing the drug release behavior from the FeHA nanoparticles [30,31,32]. Model parameters and goodness of fit are reported in Table 5. In this regard, KP model is described by the following equation:

(1) $$\frac{M_{t}}{M_{\infty }}=\;Kt^{n}$$

where *M_t_/M*_ꝏ_ represent the released fraction of payload at time *t*, while *K* and *n* are the rate constant and release exponent of the Korsmeyer-Peppas model, respectively. This semi-empirical model is commonly used to describe release from matrixes exhibiting non-Fickian transport, where both diffusion and advection contribute to release. When *n* = 0.5, the release follows Fickian diffusion, when *n* < 0.5, the release is quasi-Fickian, when 0.5 < *n* < 1, the release is anomalous (non-Fickian), and when *n* = 1, the release follows zero-order kinetics [33]. KP semi-empirical model is particularly suited for systems with an exponential release pattern in the early phases of drug diffusion. However, it is important to recognize that the KP model is valid only for roughly 60% of the total drug release [33]. Beyond this level, the foundational assumptions of the model lack significance, resulting in a loss in mechanism predictability. Moreover, for delivery systems with a sigmoidal release profile, namely those with an accelerating and then decelerating release rate, different modeling methodologies are required. The Weibull model proves to be a more adaptable and thorough empirical model in these instances. In contrast to KP model, the Weibull function may describe a diverse array of release behaviors, encompassing both exponential and sigmoidal curves, hence rendering it suitable for systems exhibiting more intricate kinetic patterns. Weibull model can be described by the following equation:

(2) $$\frac{M_{t}}{M_{\infty }}=\;1-exp(-\left({Kt)}^{\beta }\right)\;$$

where *M_t_*/*M*_ꝏ_ represent the released fraction of payload at time *t*, *K* is the scale constant related to the release rate, and d is the shape parameter that characterizes the release curve shape. Parameter *β* indicates the nature of the release kinetics, with *β* ≤ 0.75 signifying a parabolic release curve governed by Fickian diffusion, 0.75 < *β* < 1 implying a mixed process, *β* = 1 corresponding to exponential release, and *β* > 1 corresponding to a sigmoidal release pattern (characterized by a lag phase followed by accelerated release). The *β* parameter in the Weibull model can be related to the *n* exponent in the KP model, providing a connection between the two models [34].

In the present work, the siRNA release profiles from FeHA NPs exhibit unique kinetic characteristics, as demonstrated by their respective curve forms and model fits. The fitting of data with the Weibull model elucidates the mechanical distinctions between the two systems: Cit-FeHA-Sh demonstrates an exponential release profile characterized by a shape parameter *β* < 1, signifying Fickian diffusion-dominated release, wherein the rate diminishes over time due to concentration gradients. Conversely, Cit-FeHA-Sh exhibits a sigmoidal release pattern, with a *β* > 1 indicating a more intricate, multi-phase mechanism, presumably requiring an initial diffusion, succeeded by matrix reconfiguration or degradation-facilitated release [34]. The mechanistic observations align with the variations in siRNA loading; increased loading as the one seen in Cit-FeHA-Sl, may have resulted in a relatively higher tendency in aggregation, which hindered considerably the initial siRNA release. Subsequently, the breakdown of such agglomerates, leading to NP degradation, could have facilitated siRNA release in later stages, hence contributing to the observed sigmoidal release profile. Moreover, the KP model showed a strong correlation only with the Cit-FeHA-Sh formulation, with the fitted *n* value of the KP model substantially corresponding with the *β* value of the Weibull model (*β* < *0.5*), indicating that both models represent a comparable quasi-Fickian, diffusion-controlled release. Nevertheless, the KP model inadequately characterizes the Cit-FeHA-Sl sample, reinforcing the notion that its intricate, non-Fickian behavior surpasses the model’s empirical limitations. Ultimately, these results highlight the significant impact of FeHA NP composition and siRNA loading on release kinetics while also demonstrating the effectiveness of the Weibull model in precisely characterizing both straightforward and intricate release behaviors in hydroxyapatite-based siRNA delivery systems.

## 3. Materials and Methods

### 3.1. Materials

Calcium hydroxide (Ca(OH)_2_, 96% pure), iron (II) chloride tetrahydrate (FeCl_2_·4H_2_O, ≥99.0% pure), iron (III) chloride hexahydrate (FeCl_3_·6H_2_O, ≥98.0% pure), orthophosphoric acid (H_3_PO_4_, ≥85% pure), sodium citrate tribasic dihydrate (Na_3_(C_6_H_5_O_7_)∙2H_2_O, ≥99.0% pure), o-phenanthroline (1,10-phenanthroline, ≥99% pure), citric acid (C_6_H_8_O_7_) (≥99.5%), and hydrochloric acid (HCl, 37% pure) were purchased from Sigma Aldrich (St. Luis, MO, USA). Model siRNA (Silencer™ GAPDH Positive Control siRNA, in vivo ready) and RNAse-free water were purchased from Thermo Fisher Scientific (Waltham, MA, USA). All reagents were used without further purification. Solutions were prepared with ultrapure water (18.2 MΩ × cm, 25 °C, Arium© pro, Sartorius, Gottingen, Germany).

### 3.2. FeHA NPs Synthesis and Citrate Functionalization

FeHA NP synthesis was based on the work of Iannotti et al. [15]. Briefly, an H_3_PO_4_ aqueous solution (0.7 M, 75 mL) was added dropwise into an aqueous solution of Ca(OH)_2_ (0.79 M, 100 mL) under constant stirring and heating. Simultaneously to phosphoric acid addition, aqueous solutions of FeCl_2_ (0.37 M, 20 mL) and FeCl_3_ (0.37 M, 20 mL) were also added using the same dropping rate. The addition of H_3_PO_4_ and Fe^2+^/Fe^3+^ ions was controlled precisely by a Watson-Marlow 120S/DV peristaltic pump (Falmouth, Cornwall, TR11 4RU, UK). The Fe/Ca molar ratio was set to 20 mol %, and the Fe(III)/Fe(II) ratio was 1. Afterward, the solution was kept under heating and constant stirring for 3 h, then left to age at room temperature overnight. Several reaction conditions were varied to optimize FeHA NPs properties, namely reaction pH (6 and 9), temperature (37°C and 45 °C), and acid/iron addition rate (3 and 30 mL/min). Reaction pH was checked using Whatman^®^ indicator papers and corrected with 1 M sodium hydroxide (NaOH, Sigma Aldrich, ≥98.0% purity). After maturation, the obtained FeHA NPs were recovered by centrifugation (12,000 rpm, 5 min) and extensively washed with water 4 times, followed by freeze-drying. After synthesis and purification, an aliquot was freeze-dried while the remaining material was redispersed in water at a concentration of 30 mg/mL and stored at 4 °C until further use.

As a control, non-doped HA was also prepared. Briefly, an H_3_PO_4_ aqueous solution (0.7 M, 75 mL) was added dropwise with the use of a peristaltic pump into an aqueous solution of Ca(OH)_2_ (0.79 M, 100 mL) under constant stirring and heating at 45 °C.

To improve the colloidal stability of FeHA NPs they were functionalized with citrate (Cit-FeHA NPs). Sodium citrate solution (0.1 M, 25 mL) was added into the FeHA NPs suspension (final pH = 9). The suspension was sonicated with a VCX500 tip sonicator (Sonics & Materials, Inc., Newtown, CT, USA) in an ice bath. Sonication parameters were 30% amplitude, 15 min, and a 10 s sonication/10 s pause cycle. Afterward, Cit-FeHA NPs were recovered by centrifugation (12,000 rpm, 5 min) and washed with water to remove residual citrate. Cit-FeHA NPs were resuspended in water at a concentration of 30 mg/mL and stored at 4 °C, while an aliquot was freeze-dried for characterization.

### 3.3. FeHA and Cit-FeHA Characterization

X-Ray Powder Diffraction (XRPD). PXRD patterns of the dry samples were recorded on a D8 Advance diffractometer (Bruker, Karlsruhe, Germany) equipped with a Lynx-eye position sensitive detector in Bragg–Brentano geometry. Cu Kα radiation (*λ* = 1.54178 Å) generated at 40 kV and 40 mA was used. PXRD patterns were recorded in the 10–80° (2*θ*) angular range with a counting time of 0.5 s and a step size of 0.02°. Semiquantitative phase analysis was made by full profile analysis of XRD spectra (TOPAS 5, Bruker, Karlsruhe, Germany).

Fourier-Transform IR Spectroscopy (FTIR). FTIR spectra of dry samples were collected in attenuated total reflectance (ATR) mode with a Nicolet iS5 spectrometer (Thermo Fisher Scientific Inc., Waltham, MA, USA) using an iD7 diamond ATR accessory. The spectra were collected with a resolution of 4 cm^−1^ by the accumulation of 32 scans covering the 4000 to 400 cm^−1^ spectral range.

Field-Emission Scanning Electron Microscopy (FE-SEM). Micrographs of the samples were collected with a ZEISS ΣIGMA microscope (ZEISS NTS GmbH, Oberkochen, Germany), operating at a 4 kV acceleration voltage, with a working distance of 3.5 mm, and acquired using the InLens detector at 150,000× magnification. FeHA NPs were diluted with ultrapure water to a concentration of 0.1 mg mL^−1^. Afterward, a drop of NP suspension was deposited on a flat, mirror-polished silicon wafer mounted on an aluminum stub and dried at room temperature. Once the samples were dried, they were sputter-coated (Polaron E5100, Polaron Equipment, Watford, Hertfordshire, UK) with 2 nm of Pt/Pd (80:20) alloy to provide electrical conductance.

Inductively coupled plasma optical emission spectrometry (ICP-OES). Quantification of Ca, P, and Fe was performed with an Agilent 5100 instrument (Agilent Technologies, Santa Clara, CA, USA). Before analysis, 10 mg of dry sample was dissolved in 50 mL of 2 wt.% HNO_3_ solution in triplicate.

UV-visible spectroscopy (UV-Vis). Quantification of Fe^2+^ content of the samples was performed through a colorimetric method involving o-phenanthroline. In a pH range of 4–5, ferrous ions react with o-phenanthroline to form the stable red-orange complex [(C_12_H_8_N_2_)_3_Fe]^2+^, which is detectable at 510 nm via UV-visible spectrophotometry. Then, 20 mg of FeHA powder was dissolved in 1 mL of 0.5 M HCl after confirming that hydrochloric acid did not affect the concentration of the Fe^2+^-complexed compound, at least for the duration of the analysis. 10 mL of sodium citrate–citric acid buffer (0.1 M, pH 4) was added to the sample solution to maintain the pH at approximately 4–5 and to prevent the oxidation of Fe^2+^. Subsequently, 5 mL of 0.2 wt.% o-phenanthroline solution was added to reach a nominal Fe^2+^/o-phenanthroline molar ratio of 1/3. Afterward, the solution was diluted up to 50 mL with ultrapure water. The Uv-Vis spectra of the obtained solution were recorded with a Lambda 35 UV/vis spectrometer (PerkinElmer Instruments, Waltham, MA, USA). The difference between the total quantity of Fe (determined by ICP) and the amount of Fe^2+^ (determined by UV-vis) was used to calculate the amount of Fe^3+^.

Thermogravimetric analysis (TGA). Analyses were performed using an STA 449C Jupiter (Netzsch GmbH, Selb, Germany) apparatus. 10 mg of dry sample was weighed in an alumina crucible and heated from room temperature to 1100 °C under air flow with a heating rate of 10 °C/min. 

Specific Surface Area (SSA_BET_). SSA_BET_ was measured through N_2_ gas adsorption method using a Surfer instrument (Thermo Fisher Scientific Inc., Waltham, MA, USA) and Brunauer–Emmett–Teller method. Before measurement, samples were degassed at 200 °C for 3 h under vacuum.

Magnetic characterization. The samples were characterized by measuring hysteresis loops by Alternating Gradient Force Magnetometry (AGFM) at room temperature. For each analysis, ultra-thin plastic substrates of about 3 × 3 mm^2^ were used; a small amount of powder (0.20–0.40 mg) was deposited on these, which was then fixed to the substrate with a small amount of cyanoacrylate. The magnetization curve was measured for each sample in hysteresis loop mode (from +μ_0_H_max_ to −μ_0_H_max_ and then from −μ_0_H_max_ to +μ_0_H_max_) in a maximum magnetic field μ_0_H_max_ equal to 2 T and with a field variation step equal to 100 Oe. To obtain the final graphs, the following were subtracted from the measured magnetic moment: the diamagnetic contribution of the measuring probe, that of the plastic substrate, and that of the cyanoacrylate. The subtraction occurred after having appropriately re-proportioned the last two contributions with respect to the sample weight, taking into account the relative weights of the sample, substrate, and cyanoacrylate in each prepared sample. Starting from the measured magnetic moment, the magnetization of the sample in Am^2^/kg was obtained using the weight of the sample. The measuring probe (parallel probe) was calibrated at the beginning of each measurement day with a known time standard (ultrapure Pt disk).

Dynamic Light Scattering (DLS) and electrophoretic mobility. The measurement of nanoparticles’ hydrodynamic diameter distribution and electrophoretic mobility (ζ-potential) was performed using a Zetasizer Nano ZSP instrument (Malvern Instruments, Malvern, UK). Samples were analyzed in suspension at 1 mg mL^−1^ concentration and at pH 7. The hydrodynamic diameter distribution of the samples at 25 °C was measured using hydroxyapatite and water refractive indexes (1.63 and 1.33) as working parameters for the samples and the solvent, respectively, for three measurements of at least 10 runs. Particle size is reported as the Z-average of hydrodynamic diameter distribution. ζ-potentials were quantified as the electrophoretic mobility at 25 °C of three separate measurements (maximum 100 runs each) by laser Doppler velocimetry using a disposable electrophoretic cell (DTS1061, Malvern Ltd., Worcestershire, UK) with the same sample and solvent parameters.

### 3.4. In Vitro Cyocompatibility Assay

Cell Culture. WS1 human skin fibroblast cells (ATCC CRL-1502), purchased from American Type Culture Collection (ATCC) were cultured in complete cell culture medium consisting of α-MEM supplemented with 10% fetal bovine serum (FBS) and 1% penicillin-streptomycin (Pen/Strep, 100 U/mL–100 μg/mL). Cells were maintained in 75 cm^2^ culture flasks at 37 °C in a humid atmosphere with 5% CO_2_. Once the cells reached approximately 80% confluency, they were detached using 3 mL of 10× trypsin in PBS (1:9) for 3 min and subsequently centrifuged at 1000 rpm for 5 min and resuspended in fresh media. Cell number and viability were assessed with the trypan-blue dye exclusion test. For cytocompatibility tests, cells were seeded at a density of ~12,500 cells/cm^2^. The day after different concentrations of cit-FeHA nanoparticles (50 µg/mL, 20 µg/mL, 10 µg/mL, and 5 µg/mL) were added to the cell culture by dilution in the media. Nanoparticles’ suspension in the media was vortexed before being added to the cells.

Cell viability analysis. Cell viability was evaluated utilizing the MTT assay [35]. The MTT reagent (3-(4,5-dimethylthiazol-2-yl)-2,5-diphenyltetrazolium bromide) was prepared at a concentration of 5 mg/mL in 1× phosphate-buffered saline (PBS). Cells were seeded in 96-well plates and incubated with the MTT reagent at a 1:10 ratio to cell culture media for 2 h at 37 °C. After incubation, the medium was discarded, and the resultant formazan crystals were solubilized in 1 mL of dimethyl sulfoxide (DMSO) for 15 min while stirring. Only metabolically active (viable) cells can reduce MTT to formazan, resulting in a pinkish solution when dissolved in DMSO. The absorbance of this solution was quantified at 570 nm utilizing a Multiskan FC Microplate Photometer (Thermo Scientific). The absorbance values are directly correlated with the quantity of viable cells. Cell viability was quantified as a percentage in comparison to untreated control cells. All samples were analyzed at days 1 and 5 in quadruplicate (N = 4).

Statistical analysis. Results of the MTT assay were expressed as mean value ± standard deviation (SD) plotted on the graphs. Statistical analysis was performed by two-way ANOVA, followed by Bonferroni’s post hoc test using GraphPad Prism software (version 8.0), with statistical significance set at *p* ≤ 0.05.

### 3.5. GAPDH siRNA Adsorption on Cit-FeHA

Prior to adsorption, freeze-dried GAPDH siRNA was dissolved in RNAse-free water to prepare a 100 µM stock solution. Cit-FeHA suspension was diluted up to either 1 or 4 mg/mL (5 mL), and 50 µL of siRNA solution was added to obtain a final concentration of 1 µM. The suspension was incubated for 1 h at 37 °C to obtain siRNA-Cit-FeHA NPs. Hereinafter, we code the Cit-FeHA suspensions obtained at 1 or 4 mg/mL as Cit-FeHA-Sh and Cit-FeHA-Lh, respectively. To remove non-adsorbed siRNA, the suspension was then centrifuged at 5000 rpm for 2 min, removing the supernatant, resuspending the pellet in RNAse-free water, and then freeze-drying.

### 3.6. Characterization of siRNA-Cit-FeHA and siRNA Release

siRNA quantification. Adsorbed siRNA was measured indirectly by quantification of the non-adsorbed fraction in the supernatant. siRNA quantification was performed by using the Quant-it microRNA assay kit (Thermo Fisher Scientific Inc., Waltham, MA, USA) according to the manufacturer’s instructions. A calibration curve of siRNA between 0 and 1 µM was used. Fluorescence intensity was measured with a Fluoroskan Microplate Fluorometer (Thermo Fisher Scientific Inc., Waltham, MA, USA) at 485/538 nm excitation/emission wavelengths. Three replicates were performed for each sample. Adsorbed siRNA is expressed both as adsorption efficiency, i.e., weight percentage relative to used siRNA mass, and as payload, i.e., mol/mg relative to the mass of Cit-FeHA NPs.s

siRNA-Cit-FeHA NPs characterization. siRNA-loaded nanoparticles were characterized as dry powders by FE-SEM, FTIR, and DLS, as reported above.

siRNA release from siRNA-Cit-FeHA NPs. A total of either 5 mg or 20 mg of siRNA-Cit-FeHA was dispersed into 5 mL of RNase-free water at a final concentration of 1 mg/mL and 4 mg/mL, respectively, in triplicate. The suspension was maintained at 37 °C under horizontal shaking. At scheduled times (5 h, 1 day, and 1, 2, 3, 4, 5, and 6 weeks) NPs were separated from the liquid phase by centrifugation (5000 rpm, 2 min), and 200 µL of the supernatant was removed for siRNA quantification as reported above. After that, 200 µL of fresh RNase-free water was added, and the pellet was resuspended by vortexing gently at low speed, and the suspension was again put under agitation until the subsequent timepoint.

To assess the drug release kinetics, experimental data were analyzed through nonlinear least-squares regression utilizing OriginPro 8.1 (OriginLab Corp., Northampton, MA, USA). Model parameters were estimated through the minimization of the residual sum of squares (RSS), while the goodness-of-fit was evaluated using the correlation coefficient R^2^ (COD) values.

## 4. Conclusions

In this work we have explored FeHA and Cit-FeHA NPs as potential siRNA delivery agents. Our data proved the successful synthesis of these NPs possessing magnetic susceptibility. The addition of citrate did not only improve FeHA colloidal stability but also allowed the efficient removal of magnetite impurities from the product. We demonstrated that the Cit-FeHA NPs surface can be readily functionalized with a model therapeutic siRNA through electrostatic interaction, adsorbing the siRNA quickly with almost 90% efficiency and preventing its degradation. SiRNA-Cit-FeHA NPs release their payload with a slow and controlled release over several weeks, allowing a controlled release in response to local pH changes or external magnetic stimuli. Altogether, in this work we prove the high potentiality of FeHA to be applied as gene delivery nanomaterials, representing a promising theranostic platform combining bioinspired materials, genetic nanomedicine, and magnetic drug delivery.

## Figures and Tables

**Table 1:** Chemical composition and specific surface area of the samples.

Sample	Ca (wt.%) ^a^	P (wt.%) ^a^	Fe_tot_ (wt.%) ^a^	Fe^2+^ in HA (wt.%) ^b^	Fe^3+^/Fe^2+^ in HA (wt.%) ^b^	Ca/P (mol) ^a^	(Ca+Fe_HA_)/P (mol) ^a^	SSA_BET_ (m^2^/g)
HA	35 ± 1	15.6 ± 0.2	-	-	-	1.62 ± 0.01	1.62 ± 0.01	97 ± 10
FeHA	44 ± 1	22.9 ± 2.5	13.0 ± 1.8	0.74 ± 0.01	10.6	1.47 ± 0.01	1.69 ± 0.02	102 ± 10
Cit-FeHA	30 ± 1	14.9 ± 0.6	7.0 ± 0.3	0.71 ± 0.02	8.8	1.53 ± 0.03	1.66 ± 0.05	204 ± 20

^a^ From ICP-OES analysis. ^b^ From UV-Vis.

**Table 2:** The content of volatile species in HA, FeHA, and Cit-FeHA by TGA analysis.

Sample	Water Loss (20–250 °C) (wt.%)	Citrate Loss (250–500 °C) (wt.%)	Carbonate Loss (600–1100 °C) (wt.%)	Total Loss (20–1100 °C) (wt.%)
HA	5.1 ± 0.5	N/A	2.0 ± 0.3	7.1 ± 0.7
FeHA	5.0 ± 0.5	N/A	2.6 ± 0.3	7.5 ± 0.8
Cit-FeHA	7.6 ± 0.8	3.1 ± 0.3	0.8 ± 0.1	11.5 ± 1.0

**Table 3:** Hydrodynamic diameter and surface charge of the samples in suspension.

Sample	Length (nm)	Width (nm)	Aspect Ratio	Z-Average (nm)	PdI	ζ-Potential (mV)
HA	177 ± 5	37 ± 1	4.6 ± 0.2	220 ± 3	0.20 ± 0.01	−18 ± 2
FeHA	186 ± 3	40 ± 1	4.8 ± 0.1	195 ± 1	0.20 ± 0.03	−24 ± 1
Cit-FeHA	180 ± 2	43 ± 1	4.0 ± 0.1	170 ± 2	0.12 ± 0.01	−35 ± 2

**Table 4:** Adsorbed siRNA content and colloidal suspension parameters of siRNA-Cit-FeHA samples.

Sample	siRNA Payload (nmol/mg)	siRNA Adsorption Efficiency (%)	Z-Average (nm)	PdI	ζ-Potential (mV)
Cit-FeHA-Sl	0.59 ± 0.02	87.0 ± 0.5	188 ± 1	0.20 ± 0.01	−33 ± 3
Cit-FeHA-Sh	0.18 ± 0.01	82.0 ± 0.2	179 ± 3	0.25 ± 0.05	−34 ± 5

**Table 5:** Fitting parameters using Weibull and Korsmeyer–Peppas model for siRNA release from FeHA.

Sample	*K_KP_* (Release Constant)	*n* (Release Exponent)	R^2^ (COD)	*K_W_* (Release Constant)	*β* (Release Exponent)	R^2^ (COD)
Cit-FeHA Sl	0.07 ± 0.03	0.4 ± 0.1	0.6373	0.053 ± 0.003	4.8 ± 0.9	0.9895
Cit-FeHA Sh	0.18 ± 0.01	0.38 ± 0.01	0.9973	0.002 ± 0.001	0.39 ± 0.07	0.9973

## Data Availability

The data will be made available on request.

## Supplementary Materials

The following supporting information can be downloaded at: https://www.mdpi.com/article/10.3390/ijms26167712/s1.
