# Supplementary material for: Iron-Doped Hydroxyapatite Nanoparticles for Magnetic Guided siRNA Delivery
Source: Int J Mol Sci. 2025 Aug 9;26(16):7712. doi: 10.3390/ijms26167712 (PMC12386263; doi:10.3390/ijms26167712)
Supplement: Supplementary file 1 [file ijms-26-07712-s001.zip › Description videos.pdf]

movie FeHA-cit in water:

Short movie showing the FeHA-Cit nanoparticles dispersed in a petri dish in doubled distilled water, in brownish colour, which follow the movement of a magnet (M = 1.2 T) placed at the bottom of the petri dish

movie FeHA-cit in cell solution:

Short movie showing the FeHA-Cit nanoparticles dispersed in a petri dish in Mesenchymal Stem Cell Growth solution (ATCC Primary Cell Solutions), in brownish colour, which follow the movement of a magnet (M = 1.2 T) placed at the bottom of the petri dish
