# Supplementary material for: Iron-Doped Hydroxyapatite Nanoparticles for Magnetic Guided siRNA Delivery
Source: Int J Mol Sci. 2025 Aug 9;26(16):7712. doi: 10.3390/ijms26167712 (PMC12386263; doi:10.3390/ijms26167712)
Supplement: Supplementary file 1 [file ijms-26-07712-s001.zip › Supplementary information.pdf]

# SUPPLEMENTARY INFORMATIONS

# Iron-Doped Hydroxyapatite Nanoparticles for Magnetic Guided siRNA Delivery

**Hina Inam [1,2,†], Lorenzo Degli Esposti [3,†], Federico Pupilli [1], Marta Tavoni [1], Francesca Casoli [4], Simone Sprio [1,*] and Anna Tampieri [1,*]**

[1] Institute of Science, Technology and Sustainability for Ceramics (ISSMC), National Research Council of Italy (CNR), 48018 Faenza, Italy
[2] Department of Material Science and Technology, University of Parma, 43121 Parma, Italy
[3] Dipartimento di Chimica e Chimica Industriale, Università degli Studi di Genova, 16146 Genova, Italy
[4] Institute of Materials for Electronics and Magnetism (IMEM), National Research Council of Italy (CNR), 43124 Parma, Italy
* Correspondence: simone.sprio@issmc.cnr.it (S.S.); anna.tampieri@issmc.cnr.it (A.T.)
† These authors contributed equally to this work.

1. **Methodology**

*Structural and dimensional analyses*

Cell parameters and crystallite size were assessed by full profile analysis of the XRD spectra (TOPAS 5, Bruker, Karlsruhe, Germany). The splitting factors were calculated from FTIR spectra by dividing the sum of the absorbance of the peaks at 562 $cm^{-1}$ and 602 $cm^{-1}$ from $v_4(PO_4)$ bond bending by the absorbance of the minimum between these two peaks.

To obtain NPs dimensional analysis, ImageJ was used to analyse grayscale SEM images in TIFF format using the following sequential steps: Set Scale, Brightness/Contrast, and Threshold. The width and length of NPs were measured then the aspect ratio was calculated as the ratio between the length and width of each NR. 100 NPs were measured to provide data with suitable statistical significance. Subsequently, length and width data were furtherly elaborated using the ORIGIN 8.1 software. Gaussian fitting of the size distribution was used to determine the mean width, length and aspect ratio.

2. **Results**

**Table S1**. Crystal data on the synthesized apatites

| | **a (Å)** | **c (Å)** | **Vol (Å$^3$)** | **c/a** | **D (nm)** | **Splitting Factor** |
|---|---|---|---|---|---|---|
| | | | | | | |
| **Pure HA** | 9.428073±0.001078 | 6.894879±0.000803 | 530.8±0.1 | 0.731 | 15±2 | 2.48 |
| **Fe-HA** | 9.428243±0.001794 | 6.881462±0.001293 | 529.7±0.2 | 0.730 | 17±6 | 2.48 |
| **Cit-FeHA** | 9.415808±0.001911 | 6.893973±0.001391 | 529.3±0.2 | 0.732 | 16±5 | 2.63 |

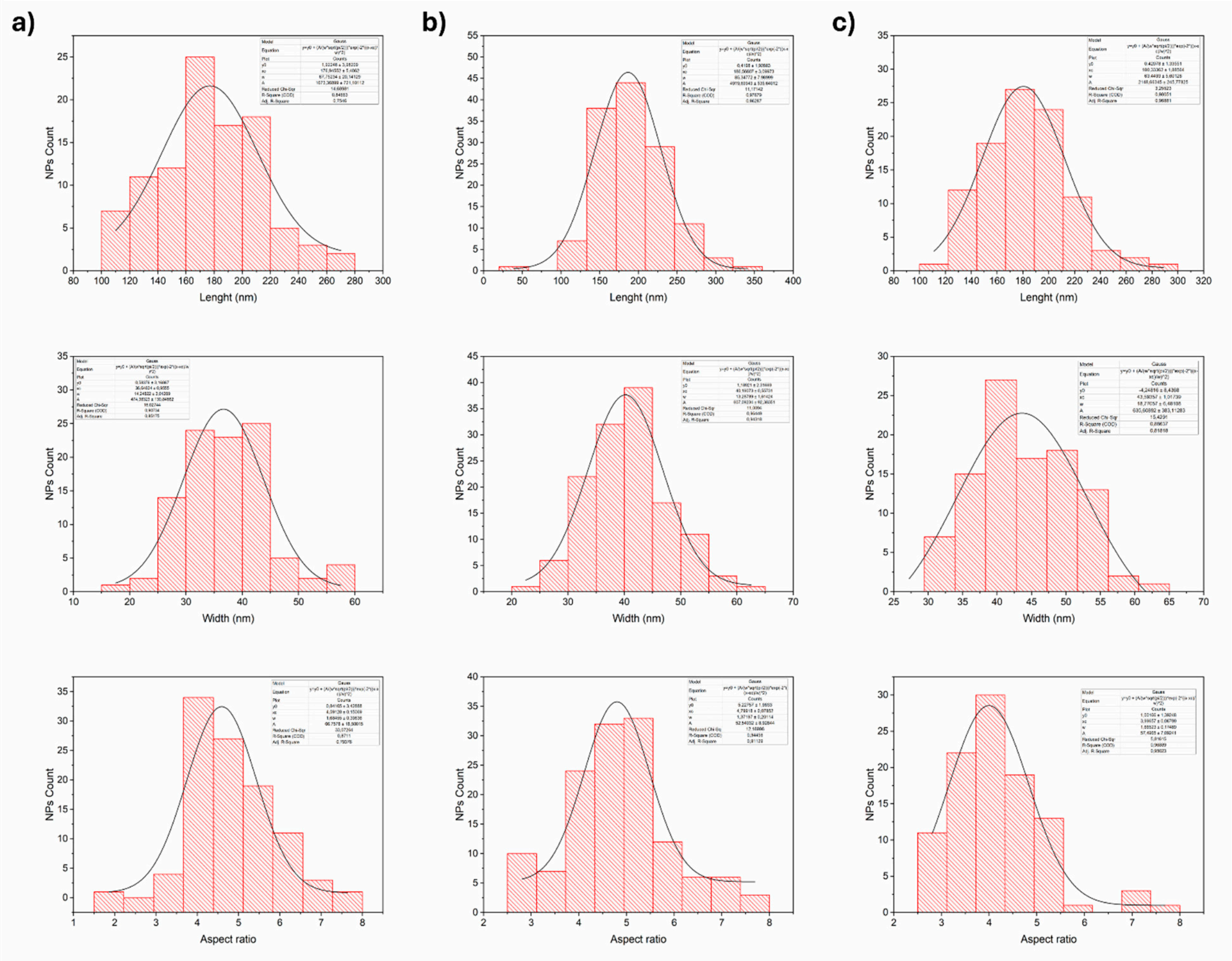


**Figure S1:** Dimensional analysis of length, width and aspect ratio calculated from SEM micrographs of HA (**a**), FeHA (**b**) and Cit-FeHA (**c**) NPs.

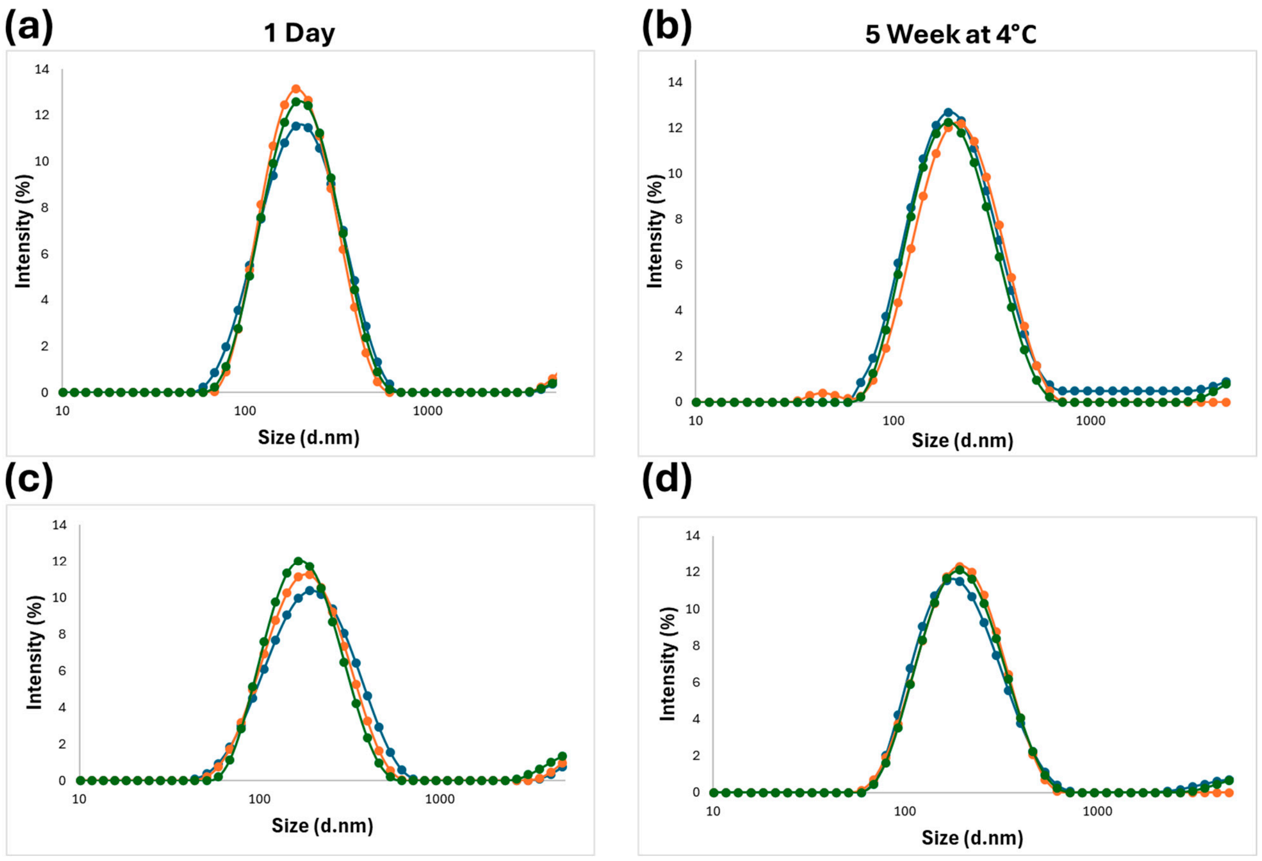


**Figure S2:** DLS size distribution profiles of Sl FeHA at day 1 (a) and after 5 weeks (b), Sh FeHA at day 1 (c) and after 5 weeks (d) of storage.
